# Causes and Pattern of Chest Trauma Among Adults: A Scoping Review of Studies From the Middle East

**DOI:** 10.7759/cureus.49980

**Published:** 2023-12-05

**Authors:** Reem S AlSulaiman, Safia M Al Abbas, Zahra A Alshaikh, Ghadeer S Almoallem, Fatimah A AlOqayli, Laila O Alibrahim, Layla A Abu Abdullah, Hatem Y Elbawab

**Affiliations:** 1 College of Medicine, Imam Abdulrahman Bin Faisal University, King Fahad Hospital of the University, Al Khobar, SAU; 2 Surgery/Thoracic Surgery, Imam Abdulrahman Bin Faisal University, King Fahad Hospital of the University, Al Khobar, SAU

**Keywords:** middle east, pattern of injury, mortality, manner of injury, thoracic injury, chest trauma

## Abstract

Chest trauma incidence is increasing worldwide, and it requires attention as it is a major cause of morbidity and mortality. Worldwide, chest trauma is the second most common cause of mortality and a major cause of disability and hospitalization. Our main aim is to systematically review the prevalence, pattern, causes, manner, morbidity, and mortality of chest trauma in the Middle East among adults. This scoping review was conducted in accordance with the Preferred Reporting Items for Systematic Reviews and Meta-Analyses (PRISMA) guidelines. Screening of the relevant articles was done by using databases, including PubMed, Scopus, and Web of Science. A total of 128 articles were found as a result of searching the databases and reviewing the reference lists. Finally, nine articles met the inclusion criteria. Most of the victims were males, as reported by all studies in this systemic review. The most common cause of chest trauma was road traffic accident (RTA), as described in seven out of the nine included studies. The pattern of chest trauma included pneumothorax, hemothorax, hemopneumothorax, lung contusion, flail chest, rib fracture, and diaphragmatic injury. The rate of mortality and morbidity following chest trauma varied among the studies. However, most of the studies revealed higher rates of morbidity than mortality. Chest trauma carries economic and social burdens, and it is a serious issue, especially in males in the second to third decades. Preventive measures should be considered to decrease the prevalence of chest trauma and its related complications.

## Introduction and background

Chest trauma is considered one of the major causes of mortality and morbidity worldwide, and it is the second most common cause of mortality after head trauma. In addition, it is one of the major causes of long-term disabilities and hospitalization, which accounts for 10% of hospital admissions and 25% of trauma-related deaths. There are a variety of chest trauma causes, but the most common cause is road traffic accidents (RTA), which account for 70% of all chest trauma cases [1].

In the Middle East, chest trauma due to RTA is one of the leading causes of morbidity and mortality. Two large series from Turkey showed that blunt injury due to RTA represents two-thirds of chest traumas. Moreover, mortality due to chest trauma was 9.3%. A report from Jordan showed that RTA accounts for 77% of chest trauma cases. Furthermore, the mortality was 8.8% [2]. The Kingdom of Saudi Arabia (KSA) has the highest incidence of RTA among all international accidents. It has recorded 611,000 injuries and 86,000 mortalities due to RTA in the last two decades [1]. The most common causes of trauma in KSA are RTA and falls, and 21% of the victims were hospitalized due to chest trauma [3].

Chest trauma could be blunt or penetrating injuries. Blunt chest injuries are considered more common than penetrating injuries, which are most commonly caused by RTA, falls, and crush injuries [4]. In addition, blunt chest trauma is mostly associated with multiorgan damage, which leads to poor patient outcomes [5]. Chest trauma varies in severity from a simple rib fracture to vital organ damage, including flail chest, lung contusion, broncho-pleural fistula, cardiac tamponade, pneumothorax, hemothorax, and tracheobronchial rupture [1]. It can be divided into different types depending on the location of the injury, including lungs, chest wall, heart, major vessels, and esophagus [6].

According to the Advanced Trauma Life Support (ATLS) protocol, these injuries including airway obstruction, flail chest, massive hemothorax, open pneumothorax, tension pneumothorax, and cardiac tamponade must be diagnosed immediately due to their fatal outcomes [3]. Moreover, there are some serious injuries that could be delayed or masked due to other injuries, including tracheobronchial rupture, pulmonary contusion, traumatic aortic disruption, blunt cardiac injury, diaphragmatic tear, and esophageal perforation. As a result of that, immediate and accurate assessments must be done for patients with potentially life-threatening chest trauma [3].

Since chest trauma is increasing worldwide among adults, a collection of all data regarding the prevalence, patterns, and mortality is needed for better preventative measures. This systemic review aims to focus on the prevalence of chest trauma in the Middle East among adults, its pattern, morbidity, and mortality.

## Review

Methods

**Figure 1:**
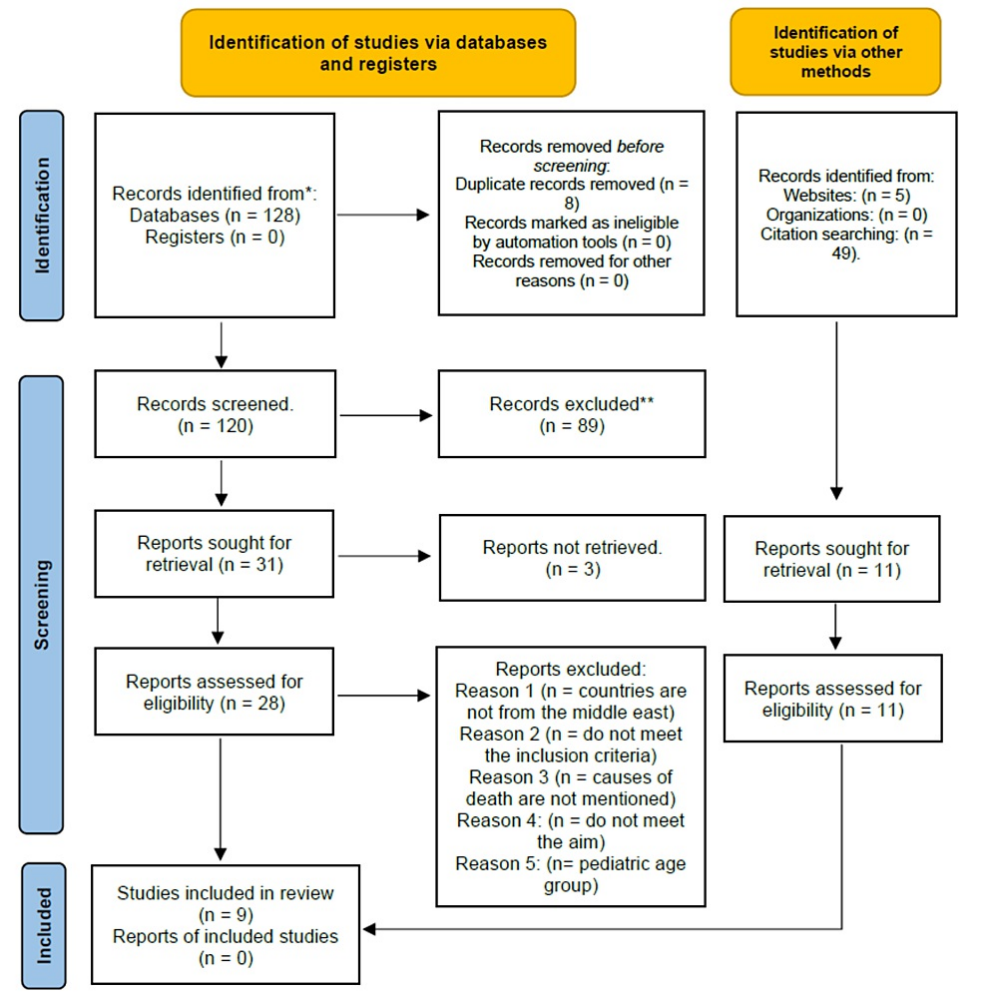
PRISMA flow diagram of study selection, which included searches of databases, registers, and other sources

This scoping review was conducted in accordance with the Preferred Reporting Items for Systematic Reviews and Meta-Analyses (PRISMA) guidelines, as presented in Figure 1 [7]. In this scoping review, we designed a search strategy to identify relevant literature. Screening of articles was done by four reviewers independently to acquire articles that focus on the prevalence, causes, patterns, morbidity, and mortality of chest trauma. Additionally, it studies the possible association between different patterns of chest trauma in relation to sex and age. This scoping review was done using PubMed, Scopus, and Web of Science. The following descriptors were used: chest, trauma, prevalence, causes, patterns, mortality, thoracic injuries, Middle East ["Bahrain", "Egypt", "Iran", "Iraq", "Israel", "Jordan", "Kuwait", "Lebanon", "Oman", "Palestine", "Qatar", "Saudi Arabia", "Syria", "Turkey", "United Arab Emirates", and "Yemen"] [8]. Boolean operators, such as ‘‘AND’’ and ‘‘OR’’, were also applied in the electronic search. The articles were assessed by two reviewers, and the studies were chosen systematically based on predetermined criteria. We included published literature about adult chest trauma in Middle Eastern countries that were published in English. Articles that are not related to chest trauma, or related to chest trauma but not for adults, not conducted in the Middle East, or not published in English were all excluded. The review was concluded on 28 January 2023.

Results

After an assessment of 120 identified studies, nine different articles from the Middle East were included in this systematic review. It highlights the different aspects of chest trauma, including age and sex, the different causes, and patterns of chest trauma. Additionally, it previews the manner of death as well as the possible outcome and percentage of the morbidity and mortality among the chest trauma cases.

Articles That Met the Criteria

In Table 1, we discuss nine articles out of 31 included articles after applying the inclusion and exclusion criteria. These nine articles highlight age and sex variation, causes, patterns, morbidity, mortality, and manner of death in chest trauma in the Middle East. The experiences of six different countries were also discussed, including one article from Iran [9], two from Turkey [10,11], one from Syria [12], one from Qatar [13], two from Saudi Arabia [3,4], and two from Egypt [14,15]. The oldest article was conducted in 2007 in Iran [7], while the newest two in 2021 in Saudi Arabia [3] and Egypt [15]. The other articles span between 2009 and 2018.

**Table 1:** Articles that met the criteria

Reference	Year	Country	Title	Journal
Zargar et al. [9]	2007	Iran	Thoracic injury: a review of 276 cases	Chinese Journal of Traumatology
Demirhan et al. [10]	2009	Turkey	Comprehensive analysis of 4205 patients with chest trauma: a 10-year experience	Interactive Cardiovascular and Thoracic Surgery
Karadayi et al. [11]	2011	Turkey	An analysis of 214 cases of rib fractures	Clinics Journal
Al-Koudmani et al. [12]	2012	Syria	Chest trauma experience over eleven-year period at Al-Mouassat University teaching hospital-Damascus: a retrospective review of 888 cases	Journal of Cardiothoracic Surgery
El-Menyar et al. [13]	2016	Qatar	Clinical presentation and time-based mortality in patients with chest injuries associated with road traffic accidents	Archives of Trauma Research
Al Mourgi et al. [3]	2016	Saudi Arabia	Prevalence and pattern of chest trauma in Saudi Arabia: a single-center experience	International Journal of Advanced Research
Alam El-Din et al. [14]	2018	Egypt	Blunt vs. penetrating chest trauma in terms of the outcome in Menoufia University Hospital	Menoufia Medical Journal
Hajjar et al. [1]	2021	Saudi Arabia	Chest trauma experience: incidence, associated factors, and outcomes among patients in Saudi Arabia	Pakistan Journal of Medical Sciences
Ibrahim et al. [15]	2021	Egypt	Retrospective statistical study of thoracic trauma patients in Al-Hussein Hospital, Al-Azhar University	The Egyptian Journal of Hospital Medicine

Relation Between Sex and Age With Chest Injuries

The nine different studies listed in Table 2 reported the variation in sex and age of chest trauma victims. Among these studies, the majority of the reported cases were male. For instance, in a retrospective study by Demirhan et al. [10], which was conducted within 10 years from 1998 to 2008 in Turkey, a total of 4,205 trauma patients with chest injuries were included in the study. The mean age of these victims was 36.2 years. In addition, most of the patients were males with a total of 3,575 out of 4205 (85%), and a total of 630 (15%) female patients [10]. Another study in Al-Mouassat University Hospital in Damascus, Syria, which was conducted over 11 years from 2000 to 2011, analyzed 888 chest trauma cases. The mean age was 31 ± 17 years. The male predominance was evident in which the number of male patients was significantly more than female patients, with a male-to-female ratio of 6.7:1 [12].

**Table 2:** Relation between sex and age with chest injuries

Reference	Age	Sex	
		Males	Females
Zargar et al. [9]	Mean (34 ± 17 Years)	246 (89.1%)	30 (10.9%)
Demirhan et al. [10]	Mean (36.2 years)	3575 (85%)	630 (15%)
Karadayi et al. [11]	Mean (51.5 years)	173 (80.8%)	41 (19.2%)
Al-Koudmani et al. [12]	Mean (31 ± 17 years)	773 (87%)	115 (13%)
El-Menyar et al. [13]	Mean (32.7 ± 15 years)	923 (92%)	81 (8%)
al Mourgi et al. [3]	21–30 years Majority	197 (87.2%)	29 (12.8%)
Alam El-Din et al. [14]	Mean (30.7 ± 17.6 years)	63 (87.5%)	9 (12.5%)
Hajjar et al. [1]	Mean (32.4 years)	206 (87.3%)	30 (12.7%)
Ibrahim et al. [15]	20-30 years are the majority	-	-

Furthermore, these studies reported a wide age variation; however, the mean age was between the second and third decades. For instance, a retrospective study conducted in Al-Taif in KSA at King Abdul-Aziz Specialist Hospital analyzed and reviewed 226 cases of chest injuries. About 12.8% of the cases were female, with a total of 29 cases, while 87.2% were male, with a total of 197 cases. The mean age was 21-30 years old [3]. However, Karadayi et al. conducted a study from 2007 to 2008, which analyzed the data retrospectively of 214 rib fracture cases. The mean age of these victims was 51.5 years, which ranges between 17 and 96 years old [11]. Further details about the age variation and sex difference were summarized in Table 2.

Causes of Chest Trauma

The different causes of chest trauma among the studies were analyzed and compared in Table 3, including RTA, falls, violence, and other unspecified causes. A study that was conducted in Saudi Arabia by Hajjar et al. [1] reported the highest percentage of RTA, which was 86.9% among the 236 participants with chest trauma. In the other studies, RTA varied between 31% and 86.7% [1]. Moreover, violence is an evident cause of chest trauma that varied among the studies between 2.2% and 42.3%. A study conducted on 100 cases by Ibrahim et al. reported 42% as the highest percentage of violence among chest trauma victims [15]. Another significant cause of chest trauma is falls, which varied between 2.5% and 23%. In a study conducted by Karadayi et al. among 214 cases, they reported the highest percentage of falls at 24.3% [11]. Additionally, Other unspecified causes of chest trauma varied between 2.5% and 27% [11].

**Table 3:** Causes of chest trauma

Reference	Sample Size (N)	RTA	Falls	Violence	Others
Zargar et al. [9]	276	137 (49.6%)	33 (12%)	89 (32.2%)	17 (6.2%)
Demirhan et al. [10]	4205	1998 (47.5%)	222 (5.2%)	1733 (42.3%)	252 (6%)
Karadayi et al. [11]	214	138 (64.5%)	52 (24.3%)	7 (3.3%)	17 (7.9%)
Al-Koudmani et al. [12]	888	292 (33%)	201 (23%)	365 (41%)	30 (3%)
El-Menyar et al. [13]	1004	771 (77%)	-	233 (23%)	
Al Mourgi et al. [3]	226	196 (86.7%)	11 (4.8 %)	16 (7.1%)	3 (1.4%)
Alam El-Din et al. [14]	100	55 (76,4%)	8 (11.1%)	-	9 (12.5%)
Hajjar et al. [1]	236	205 (86.9%)	6 (2.5%)	11 (4.7%)	14 (5.9%)
Ibrahim et al. [15]	100	31 (31%)	-	42 (42%)	27 (27%)

Patterns of Chest Trauma

The patterns of chest trauma among the different studies were analyzed and compared in Table 4. These patterns include hemothorax, pneumothorax, hemo-pneumothorax, lung contusion, rib fracture, flail chest, diaphragmatic injury, and other patterns. Hajjar et al. study reported the highest percentage of pneumothorax among the chest trauma cases with 55.5%. In other studies, pneumothorax cases varied between 9% and 49% [1]. Hemothorax is considered one of the common patterns that were reported among most of the studies in this systemic review. The highest percentage of hemothorax was 50% reported by Alam El-Din et al.'s study [14]. On the other hand, Al Mourgi et al.'s study reported only 13.7% of cases of hemothorax [3].

**Table 4:** Pattern/type of chest trauma

Table 4: Pattern/Type of Chest Trauma								
Reference	Pneumothorax	Haemothorax	Hemo-pneumothorax	Lung contusion	Flail chest	Rib fracture	Diaphragmatic injury	Others
Zargar et al. [9]	92 (24.4%)	52 (13.8%)	46 (12.2%)	-	3 (0.8%)	145 (38.6%)	12 (3.2%)	25 (7%)
Demirhan et al. [10]	1215 (28.8%)	1016 (24.1%)	467 (11.1%)	104 (2.4 %)	72 (1.7%)	1352 (32%)	76 (1.8%)	312 (7.1%)
Karadayi et al. [11]	56 (26.1%)	42 (19.6%)	-	14 (6.5%)	-	214 (100%)	-	23 (10.7%)
Al-Koudmani et al. [12]	465 (51%)	340 (38%)	-	137 (15%)	21 (2.4%)	304 (34%)	30 (3.4%)	47 (5%)
El-Menyar et al. [13]	291 (29%)	230 (23%)	-	743 (74%)	-	572 (57%)	-	20 (2%)
Al Mourgi et al. [3]	57 (25.2%)	31 (13.7%)	37 (16.4%)	133 (58.8%)	3 (1.3%)	110 (48.6%)	5 (2.2%)	123 (54.4%)
Alam El-Din et al. [14]	49 (49%)	50 (50%)	-	36 (36%)	9 (9%)	29 (29%)	-	34 (34%)
Hajjar et al. [1]	131 (55.5%)	80 (33.8%)	-	140 (59.3%)	7 (3%)	150 (63.5%)	-	45 (19.2%)
Ibrahim et al. [15]	9 (9%)	26 (26%)	-	-	-	18 (18%)	-	Open chest injury (47%)

Another significant pattern of chest trauma was lung contusion, which varied between 2.4% and 74% among chest trauma patients. El-Menyar et al., Hajjar et al., and al Mourgi et al. reported the highest three percentages of lung contusion with 74%, 59.3%, and 58.8%, respectively [1,3,13]. Flail chest showed the lowest rate compared to other patterns, ranging between 0.8% and 9%. Moreover, rib fractures were considered a significant pattern of chest trauma, which was reported among all studies in this systemic review with a rate varied from 18% to 100%. Karadayi et al.'s study reported that 100% of chest trauma cases were having rib fractures [11]. Furthermore, the percentage of diaphragmatic injury among chest trauma patients was between 1.8% and 3.4%. Other patterns were reported among all studies of this systemic review, which varied between 5% and 54.4%.

Outcome

A wide variation was evident between the rate of mortality and morbidity following chest trauma, as presented in Table 5. For instance, the highest morbidity accounted for 78.8% reported in a study at Al-Mouassat University Hospital in Damascus, Syria, where they analyzed 888 chest trauma cases over 11 years from 2000 to 2011. On the other hand, they reported the lowest rate of mortality of 1.8% [12]. Moreover, a retrospective observational analysis in Doha, Qatar, found that among 1,004 blunt chest trauma cases, morbidity is 5.2%, and mortality is 15% [13]. Furthermore, a study conducted by Zargar et al., with a total of 276 trauma patients, reported the highest mortality rate, which is 44.16% [9].

**Table 5:** Morbidity and mortality of the population

Reference	N	Morbidity	Mortality
Zargar et al. [9]	276	-	122 (44.16%)
Demirhan et al. [10]	4205	1059 (25.2%)	391 (9.3%)
Karadayi et al. [11]	214	72 (33.6%)	20 (9.4 %)
Al-Koudmani et al. [12]	888	78 (8.7%)	16 (1.8%)
El-Menyar et al. [13]	1004	52 (5.2%)	151 (15%)
Al Mourgi et al. [3]	226	12 (5.3%)	21 (9.4%)
Alam El-Din et al. [14]	100	37 (37%)	7 (6.9%)
Hajjar et al. [1]	236	-	-
Ibrahim et al. [15]	100	52 (52%)	18 (18%)

Manner of Death

In Table 6, the manner of death varied in the rate and type among the studies. El-Menyar et al. reported the highest percentage of accidental injuries at 62% [13]. Accidental injuries ranged between 42.1% and 62% among the different studies. Moreover, homicide is a significant manner of death, which was reported between 25.4% and 57.9%. Additionally, undetermined injuries, in which the manner of death was not known, were reported by Zargar et al. and Demirhan et al. as 18.9% and 7.2%, respectively [9,10]. Furthermore, unspecified injuries were reported as 100% by Karadayi et al., Al Mourgi et al., and Alam El-Din et al. [3,11,14]. Moreover, suicide was not mentioned or reported in any of the studies.

**Table 6:** Manner of death

Reference	Accidental	Homicide	Suicide	Unspecified	Undetermined
Zargar et al. [9]	68 (55.7%)	31 (25.4%)	-	-	23 (18.9%)
Demirhan et al. [10]	227 (58%)	136 (34.8%)	-	-	28 (7.2%)
Karadayi et al. [11]	-	-	-	20 (100%)	-
Al-Koudmani et al. [12]	9 (56.3%)	7 (43.7%)	-	-	-
El-Menyar et al. [13]	93 (62%)	57 (38%)	-	-	-
Al Mourgi et al. [3]	-	-	-	21 (100%)	-
Alam El-Din et al. [14]	-	-	-	7 (100%)	-
Hajjar et al. [1]	-	-	-	-	-
Ibrahim et al. [15]	8 (42.1%)	11 (57.9%)	-	-	-

Discussion

This scoping review has analyzed nine different studies of chest traumas. The characteristics of the victim, causes, pattern, morbidity, mortality, and manner of death were all explored. Chest trauma is considered the second leading cause of death after head trauma [1]. In the Middle East, the majority of chest trauma-related victims are in their second or third decade of life [3,15]. This finding is similar to that of Masuma et al. as they reported that the mean age was between 20 and 39 years [16]. While in Benhamed et al., Lundin et al., and Veysi et al., most of the victims were in their fourth decade of life [17-19]. This is considered the most economically active age group, which will affect the individuals, their families, communities, and the country. As a result of that, health education must be done to reduce the incidence and severity of chest trauma in this age group [16]. The majority of the victims were males, as reported by all studies in this systemic review. Moreover, this finding is similar to that of Masuma et al., Benhamed et al., Lundin et al., and Veysi et al. as they reported that most of the victims were males as well [16-19]. It was reported to be due to the involvement of males in risky activities, including high-speed vehicles motorcycles, violence, and fall from high places due to their jobs [16].

There are different causes of chest trauma that were reported among different studies, including RTA, falls, violence, stab wounds, gunshot wounds, and other unspecified causes. The most common cause of chest trauma is RTA, followed by falls, as reported by all studies in this systemic review. In addition, RTA accounts for 70% of all chest trauma cases [1]. Other studies, including those of Masuma et al. and Veysi et al., showed similar results [16,19]. Meanwhile, Lundin et al. showed that the most common cause of chest trauma is falls, which accounted for 24% of the cases, followed by RTA in 21% of the cases [19].

Chest trauma has a diverse pattern around the world due to numerous environmental and sociopolitical factors. Hemothorax was one of the most prevalent patterns of chest trauma in our systemic review with the highest percentage of hemothorax, which was 50% reported by El-Din et al. [14], followed by Al-Koudmani et al. [12] with 38%. Similar statistics to those from the current investigation, which found 78 (65%) patients with hemothorax, were found by Khan et al. in a study of 120 patients who suffered penetrating trauma [20]. The second pattern was rib fractures, varying from 18% and reaching 100% in Karadayi et al.'s study, which was conducted on patients who have rib fractures and other chest traumas [11]. A significant pressure striking the chest wall during RTA can result in traumatic rib fractures. Mefire et al. also studied the epidemiology of 354 cases, including both blunt and penetrating trauma, and found that the most common lesions were haemothorax at 38.7% and rib fractures at 50.3% [21]. Furthermore, other studies reported the same findings, in which rib fracture was the most prevalent pattern [22,23].

According to different studies, pulmonary contusion was found in 25%-35% of all blunt chest trauma cases [24]. In our systemic review, lung contusion was another pattern of chest trauma that was mostly prevalent in the studies of Al-Koudmani et al., El-Menyar et al., and Hajjar et al., with 74%, 58.8%, and 59.3%, respectively. This finding is similar to that of Sikander et al. in which lung contusion affected 46.3% of patients [25]. Flail chest and diaphragmatic chest injury were the least common patterns of chest trauma according to our study.

There was a large variation in the rate of mortality and morbidity following chest trauma. Most of the studies in this systemic review showed higher rates of morbidity than mortality. Zargar et al. showed the highest rate of mortality, which was 44.16% [9]. The mortality rates of Masuma et al., Benhamed et al., Lundin et al., and Veysi et al. were 21.6%, 16.2%, 6.6%, and 18.7%, respectively, which is similar to the findings in this scoping review [16-19]. In Masuma et al., there were predictors that were statistically significant and related to the mortality rate, including associated injuries, time from door to treatment, analgesia mode, presence of critical injuries, requirement of invasive ventilation, and bilateral chest involvement [16]. In Many et al., the morbidity rate was studied in the form of the development of pneumonia, the need for mechanical ventilation, and the need for admission to an intensive care unit (ICU), which account for 17.2%, 22.3%, and 9.4%, respectively. Moreover, a number of predictors of mortality rate were studied, including Glasgow coma scale (GCS) ≤ 8, SpO2 ≤ 80%, pulse > 110, injury severity score (ISS) > 16, presence of ≥ 4 rib fractures, head injury on computed tomography (CT), other systemic injuries, and the need for the use of mechanical ventilation [26].

The manner of death in fatal cases varied among the studies in this systemic review. The majority of fatal cases were accidental in nature [13]. Moreover, the homicidal manner of death was high as well among the fatal cases [12,13,15]. In addition, the undetermined manner of death was reported by some of the studies, which account for a few of the fatal cases [9-10]. There was a high rate of unspecified manner of death, which was 100% among three studies of this systemic review [3,11,14]. Suicidal manner of death was not mentioned or reported in any of the studies. Kumar et al. showed similar findings with 60.97% accidental, 38.21% homicidal, and 0.81% suicidal manner of death [27].

Limitations

Some of the general weaknesses of scoping or systematic reviews include susceptibility to bias such as in the article selection phase. Additionally, selectively reporting statically significant findings may mislead the analysis of evidence outcomes in a scoping or systematic review. Moreover, the empirical results reported in this scoping review should be considered in light of the following limitations. The first limitation is that the included articles in this scoping review did not cover all the Middle Eastern countries equally and sufficiently. That was due to a lack of previous research papers on chest trauma topics in some Middle Eastern countries. Therefore, further studies on this topic are needed. The second limitation concerns the keywords used in searching the databases; they could be expanded in order to include more studies in the review. The last limitation was due to the limited access to free full text, which resulted in their exclusion.

Furthermore, the included studies had a number of limitations. For instance, one of the studies might not be completely representative of all patterns and types of chest trauma because it was hospital-based only, in which minor cases were not included. Additionally, the mortality rate could be higher than reported, due to many severe cases where there was death at the scene, which was not reported. Moreover, some of the studies had retrospective observational study designs and small sample sizes. Moreover, the exact circumstances of RTA were not mentioned and whether the victims used a seatbelt or if they were under alcohol influence.

## Conclusions

Most of the victims were males, as reported by all studies in this systemic review. The most common cause of chest trauma was RTA, as described in seven out of the nine included studies. The pattern of chest trauma included pneumothorax, hemothorax, hemo-pneumothorax, lung contusion, flail chest, rib fracture, and diaphragmatic injury. The rate of mortality and morbidity following chest trauma varied among the studies. However, most of the studies revealed higher rates of morbidity than mortality. Chest trauma carries economic and social burden, and it is a serious issue, especially in males in the second to third decades. Preventive measures should be considered to decrease the prevalence of chest trauma and its related complications.
